# Histology of the Dental Tissues

**Published:** 1870-10

**Authors:** 


### SELECTED ARTICLES.

---

### ARTICLE VI.

### *Histology of the Dental Tissues.*

Professor Judd on Professor Noel's Reply.

In the April number of the JOURNAL, we passed in review the principal articles found in a previous number of the "American Journal of Dental Science," and among the rest one by Prof. Noel, upon the histology of the dental tissues. To some of our views, as then expressed, the author of the article referred to objects, and replies at considerable length in the June number of the "American Journal." As the subject itself is one of interest to the whole profession, and as it is important that all new doctrines should be carefully examined, we shall offer no apology to the readers of the JOURNAL, for referring again to this topic.

We do this, however, with no captious intent of defending positions, which we may have previously taken, simply because we have committed ourselves thereto, but for the purpose of arriving at the truth. The assertions by the author that "Fresh dentine has no tubuli, but has uncalcified fibrillæ solid or nearly solid," also, "that the tubuli found in the dry tooth, or dead and dry teeth, are formed by the desiccation and evaporation of these dentinal fibrillæ, and have no existence in the living tooth," were very briefly commented on, and have brought forth a rather spirited reply. The author complains that we have based our argument upon the unauthorized assumption that there are hollow rods in fresh dentine. The only difference in the author's views and our own upon this point is simply one relating to the proper nomenclature to be employed, and that, i. e., the nomenclature made use of, was what we had reference to when we referred to the *conversion* of hollow rods into fibrillæ, as doubtless our readers generally understood. We would remark, in the first place, that a hollow body is not necessarily one that has a vacuum within it, as the heart is a hollow organ, which is filled with blood; and the venous and arterial *tubes* are, nevertheless, hollow *tubes*, even if the blood in them has not been desiccated or evaporated. Now, if the fibrillæ of the dentinal organ are calcified upon their surface, whilst their central axis are fluid, or at most, soft solid, are they not tubes just as much before their contents are desiccated or evaporated, as they are after those changes have taken place, which have reference only to the contents themselves? Therefore we do not think that the "tubuli found in the dry tooth, are *formed* by the desiccation and evaporation of these dentinal fibrillæ, and have no existence in the living tooth." We prefer, therefore, to speak of dentine as made up of a connective tissue base, which, with the lime salts deposited in it, assumes the form of tubes radiating from the periphery of the pulp to the periphery of the dentine. We enumerate, also, as a part of the dentine the contents of the tubuli just as we would in describing the cementum or other bones, include its connective tissue base and salts, the contents of the lacunæ, and when Haversian canal are present, their contents, that is to say, the veins. arteries, absorbents, &c., are as much a part of a bone as is the phosphate of lime or its connective tissue; consequently we should differ again from our author in our description of dentine, as he states that "all dentine is calcified fibrillæ," whilst we consider the nuclear matter of the dentinal tube, as well as the uncalcified formed material, as parts of the dentine. But enamel and dentine are not vitalized tissues, so far as their *calcified* portions are concerned. "Enamel is dead, perfectly dead," &c. The author does not deny but that the enamel organ and the dentinal organ were vitalized structures up to the very moment that calcification took place, when they became enamel and dentine, consequently dead tissues. We would ask, "What are the evidences of their death?" There is no proof that any change in this direction in the connective tissue base of these structures has taken place at all. The salts may be removed, and the base, at least in dentine, remains in them apparently as perfect as it was before the deposition of salts took place. It was a soft tissue before and it is a soft tissue after the salts have been removed from it; and we have no reason to believe that no vital changes have taken place in this organic base whilst it has been surrounded and infiltrated with inorganic matter. But as the author has seen fit to give us his definition of a vitalized tissue as one "changing under vital forces—exhibiting the three forces of organic life, (1.) assimilation, (2.) propagation, (3.) developement"—we would simply state that we consider a tissue vital which has the power of assimilation, without any reference to its propagating or developmental powers. Now, so far as any light has been thrown upon this subject, in referance to enamel and dentine, it goes to show that nutrition does take place in the organic base of these tissues. It has not only been observed by dental practitioners that the teeth grow harder as age advances, as is universally acknowledged, but that they also, under certain circumstances, grow softer, or at least more friable. It is well known to dental practitioners that where the pulp of a tooth has been destroyed, and all "vascular and nervous supply" cut off, that the dental tissues become more friable and more liable to decay. At least we think this will accord with the opinions of a great majority of the most reliable and observing men in the profession. We do not claim for this argument the force of a demonstration upon this subject, but it is at least entitled to more consideration than a bare supposition that has no single fact to which one can point that can in any degree relieve its inherent weakness. It has not yet been demonstrated that nutritive changes do not take place in the organic bases of dentine and enamel; but, on the other hand, we think that all the light we have upon this subject would lead us to an opposite conclusion.

In reply to an assertion of ours, that constant change is stamped upon every atom of matter throughout the universe, the author says; "Every atom of matter in the universe may be under a law of 'incessant change,' but if the changes are to us inappreciable, I contend that it is not proven. * * * * I have a friend traveling in Europe; he writes to me that he saw at a cathedral a molar tooth said to have belonged to one of the apostles or bishops of the early Church, evidently some hundred years old. Now, perhaps the 'organic base' of the enamel of that tooth has been undergoing constant change for the last eighteen hundred years, or at any rate, a great number of years; but my friend failed to appreciate it, for he said it was a fine tooth, in a state of excellent preservation."

We will suppose that side by side with the "molar tooth" lay the foot of a live frog, *apparently* motionless, a microscope would have revealed therein a field of activity truly surprising—blood corpuscles rushing through veins and arteries—leucocytes creeping along the inner walls of the various vessels—pigment cells scattered along here and there, altogether forming a scene of wondrous magnificence and beauty—but his "friend" would have failed to have appreciated it. A glance by the naked eye could not have revealed these wonders. So these changes of nutrition and decomposition, although no less certain and wonderful than the changes in a frogs foot, are not usually appreciated upon a mere casual observation.

But it is objected that we have cited *sensibility* as one of the signs of vitality. After stating that enamel is "perfectly insensible," he qualifies this by stating that "if enamel ever gives evidence of sensibility, it must be in extremely rare cases, and only when the dentinal fibrillæ have been prolonged into the enamel organ, and have escaped calcification, thus giving an unbroken line from enamel to pulp." We have only to remark in relation to this that the condition described by our author above is not, according to our observation, very rare, and that it is not certain but that that very state of things always obtains. We have no doubt but that the sensitiveness of enamel is due to the extension of the dentinal tubuli with their soft contents into the enamel.

Tomes states that in a well developed tooth a certain number of the dentinal tubes will be seen to pass across the line which marks the junction of the enamel and the dentine, &c., * * but in teeth of less perfect organization, the dentinal tubes, often passing into the enamel, become suddenly dilated into comparatively large elongated cavities. As the author says, "The pain in decay is dependent upon the dentinal fibrillæ." And as we have just shown that even in well developed teeth, the *fibrillæ*, or tubes, are continued into the enamel, would we not reasonably conclude that we should sometimes have *sensitive* dentine, even if our every day's experience did not demonstrate that it is so.

But again controverting a statement that we had put forth, to the effect that sensitiveness is one of the most certain signs of vitality, wherever it exists he says: "sensitiveness is not one of the most certain signs of vitality wherever it (vitality) exists, nor wherever it (sensitiveness) exists (sentence capable of each construction, and wrong in each)."

Now, in relation to the first construction, and the only one which is here argued by our author we would say that construed in that way, it would be evident to the merest tyro that no argument could be made in its favor, so that all the labor expended in that direction was thrown away; but with the second construction, which our author acknowledges to be also legitimate, and which we intended should be placed upon it, the case is entirely reversed, no argument of any force can be made against it. In order to controvert this it must be proved that sensitiveness can exist where there is no vitality, a task which, we presume, will not be hastily undertaken.

In relation to the developement of the "dentine organ" the author says: "Simultaneously"—that is, at the same time that masses of germinal matter make their appearance upon the inner surface of the tunica reflexa—" there is found upon the external surface of the pulp, i. e., external to and resting upon the tunica propria, masses of germinal matter, which grow towards the germinal matter projecting from the surface of the tunica reflexa until they meet at the line of conjunction." It is at once evident that, according to this description, the tunica propria separates the dentine organ from the pulp. In the author's reply he says that the fibrillæ of the dentine organ blends in organic continuity with the pulp tissue. If so, what has become of the tunica propria? Now, it has been held by nearly all of the best histologists of the last twenty-five years, at least, that the dentine was formed beneath the tunica propria. Beale, however, dissents from this view, but he does not state that the dentine organ is formed by *germinal matter*, *external to and resting upon the tunica propria*, as that would not accord with his views concerning the relationship which exists between the dentine organ and the pulp, for speaking of the cellular elements of the dentine organ, he says these elongated cells are not separated from the nerves, vessels and connective tissue, and, in some instances, seem to be continuous with them. We, therefore, very naturally remarked in our first article, "that it would be new to our readers that the dentinal organ is formed external to the tunica propria," &c.—*Missouri Dental Journal.*

### BIBLIOGRAPHICAL NOTICES.

